# Measuring accessibility using gravity and radiation models

**DOI:** 10.1098/rsos.171668

**Published:** 2018-09-12

**Authors:** Duccio Piovani, Elsa Arcaute, Gabriela Uchoa, Alan Wilson, Michael Batty

**Affiliations:** 1 Centre for Advanced Spatial Analysis, University College London, 90 Tottenham Court Road, London W1T 4TJ, UK; 2 Nam.R, 4 rue Foucault, Paris 75116, France; 3 Prefeitura Municipal de Teresina, Praça Marechal Deodoro da Fonseca 860, Teresina 64000-160, Brazil; 4 The Turing Institute, 96 Euston Road, London NW1 3DB, UK

**Keywords:** gravity model, radiation model, accessibility measures, Sorensen correlation index, bus rapid transit, Teresina, Brazil

## Abstract

Since the presentation of the radiation model, much work has been done to compare its findings with those obtained from gravitational models. These comparisons always aim at measuring the accuracy with which the models reproduce the mobility described by origin–destination matrices. This has been done at different spatial scales using different datasets, and several versions of the models have been proposed to adjust to various spatial systems. However, the models, to our knowledge, have never been compared with respect to policy testing scenarios. For this reason, here we use the models to analyse the impact of the introduction of a new transportation network, a bus rapid transport system, in the city of Teresina in Brazil. We do this by measuring the estimated variation in the trip distribution, and formulate an accessibility to employment indicator for the different zones of the city. By comparing the results obtained with the two approaches, we are able to not only better assess the goodness of fit and the impact of this intervention, but also understand reasons for the systematic similarities and differences in their predictions.

## 1. Introduction

Assessing the impact of new infrastructural projects is a challenging and demanding task that requires knowledge or estimates of the mobility of the individuals living in the city. Many models have been developed to this effect [1–4], focusing on different scales of the urban system, according to the quality of the data available (for a recent review on the subject refer to [5]). Traditionally, these models allocate trips from one geographical zone to another, according to estimates of where people live and work. Infrastructure projects are then assessed following changes in accessibility which are computed from the model's predictions of the people living and working in these zones. Among the models that have been proposed over the decades, the gravitational model [4] has been one of the most widely adopted in various contexts (for example [6–13]). Depending on the information available on the demographics and mobility of the individuals, this model exists in the following different forms as an unconstrained, singly constrained, doubly constrained model and mixed constrained. Naturally, the more information available to calibrate the model, the better its performance against observed data, notwithstanding the fact that information is not always available for this purpose.

Recent years have seen a dramatic increase in available data on individuals and their urban environments, allowing researchers to test these models more effectively, thus providing more detail on the outstanding problems of human mobility. This has prompted a surge in the literature, where new models have been proposed such as the *radiation model* [14]. This model takes its inspiration from the intervening opportunities model [1] where flows are modelled without parameters and take only as input the population distribution. It produces predictions with a high degree of accuracy at the intra-county scale, hence introducing a new benchmark in the field of mobility modelling. This has triggered the interest of many researchers, and many works have appeared where its predictions are compared with those of the more traditional gravity model [15–21]. These efforts have focused on comparing the accuracy of the models in reproducing observed origin–destination matrices. As shown in [18,20,21], the main limitation of the radiation model is its inability to produce adequate outcomes at different spatial scales, which is a direct consequence of its own virtue of being parameter free. In order to overcome this limitation, several solutions have been proposed: notably in [21] the authors introduce a *normalized* version of the model in order to take into account finite size effects, while in [20] the authors propose an *extended* version of the model introducing a parameter that can be calibrated to the data. The accuracy in reproducing the observed flows via the *extended* and the *normalized* versions is often comparable to those obtained using the doubly constrained gravitational model.

The origin–destination matrices used to compare the models in previous works are extracted both through conventional datasets, e.g. mobility surveys and census data, and through unconventional datasets such as mobile phone or geo-located social media data. Very often though these matrices are outdated, incomplete or obtained by biased samples of the population [22]. Here, we take a different approach, and explore the two different models by quantifying and predicting the impact of the introduction of a new bus rapid transit (BRT) system in the city of Teresina in Brazil. In 2008, the municipality of Teresina approved its Transport and Mobility Master Plan which proposes a new system of public transport in the city. It relies on an origin–destination survey of trip making conducted in 2007, which was developed to analyse travel patterns in order to predict future scenarios [23]. Although the survey is incomplete and does not contain information on the commuters' behaviour in all zones of the city, it is representative of a true policy test scenario, providing an ideal test bed for the different models, which is not being currently explored in the literature.

Moreover, BRT systems are increasing in popularity worldwide as an alternative cost-effective investment in comparison to expensive urban rail transport projects [24]. Readers can see from http://brtdata.org/ that there are more than 206 cities which have introduced some kind of BRT system and with the number of new corridors under construction increasing steadily. Indeed, emerging economies have been seduced by the publicized BRT success from cities such as Curitiba and Bogotà [25–28], which after introducing BRT systems have experienced enhanced mobility and sustainability at an affordable price. This has seen an increase in studies of BRT proposals [29–31], and the evaluation of such systems in various cities [32,33]. With this in mind, the main goal of this paper is to use both the gravitational and the radiation models to quantify the effects of such transportation interventions, measuring the variation in accessibility and comparing the results of the two models.

## 2. The case study: the bus rapid transit implementation in Teresina

**Figure 1.**
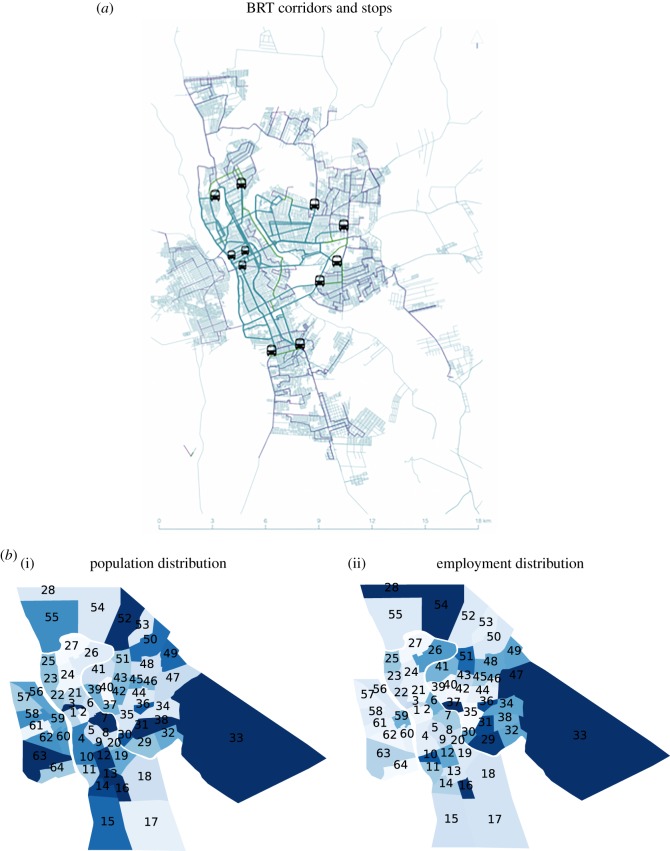
(*a*) A map of the new BRT stops and their corridors. The BRT stops are only found in Teresina but mobility in Timon is clearly affected by the scheme. (*b*) A qualitative heat map of Teresina, zones 1–55, and Timon, zones 56–64, where zones are coloured according to the population (i) and employment (ii) distribution, where the values increase from white to dark blue.

As mentioned above, our case study will be the city of Teresina in Brazil, a medium-sized metropolis which is currently implementing a BRT system. It is the capital of the state of Piauì and its metropolitan region has almost 1.2 × 10^6^ inhabitants according to the last census estimate made by the Instituto Brasileiro de Geografia e Estatística in 2015. The administrative region, named ‘Região Integrada de Desenvolvimento da Grande Teresina – RIDE/Grand Teresina’, is composed of 15 municipalities. However, only two municipalities are served by a connected urban transport network—Teresina and Timon (figure 1). Both cities together concentrate most of the population in the region with just over 10^6^ inhabitants. The metropolitan public transportation consists of a bus network, and a rail service connecting the southeast zone to the city centre. The rail service operates sharing the infrastructure with freight trains on a single track in both directions, resulting in a sparse and low usage service. For this reason, it will not be considered for cost comparisons in this study. The present structure of the public transport system is thus non-hierarchical. The majority of the lines form a radial scheme, departing from the suburbs towards the city centre with a few services that directly connect zones in the suburbs. In recent years, several exclusive bus lanes in the central area have been constructed aiming at reducing travel time on congested roads.

As previously mentioned, in 2007 the municipality of Teresina conducted an origin–destination survey which was developed to analyse travel patterns and predict future scenarios [23]. This was used for the Transport and Mobility Master Plan in 2008, which proposes a new system of public transport in the city. The original proposal suggests the implementation of a BRT system, splitting the existing routes and creating a hierarchical system of feeder, inter-terminal and trunk services. This will be composed of eight terminals connected through express bus corridors. The proposal aims to increase the effectiveness of public transportation in the city, improving the accessibility to jobs, education and public services. In this paper, through different measures of accessibilities, we evaluate the impact of such an infrastructure project.

The population and employment distribution data are provided by Teresina city council's census. The main mobility data source is the STRANS/PMT (Teresina Transport Authority) database created through an origin–destination survey conducted in 2007 including the cities of Teresina and Timon [23]. The database contains household conditions, personal socio-economic information and travel diaries per person on the day before the survey was taken. The main dataset is based on the trip data (walking times, waiting times, travel times, mode, origin and destination zones and activities and trip costs) combined with disaggregated demographic and socio-economic information about the traveller (education level, income, gender, employment, age) and traveller's household data (traffic zone, comfort and deprivation variables—number of families, bedrooms, bathrooms, sewage system, access to water, energy consumption). The survey consists of 64 traffic zones in Teresina and Timon that coincide with the cities' districts which provide the opportunity to gather socio-demographic data from the national census. In total, the dataset contains 5177 journeys distributed across 138 households. The dataset also contains information about the number of employers, students and total population in each traffic zone. Geo-referenced data are also available for bus routes and stops for all routes in Teresina and Timon and the general transit feed specification (GTFS) for the public transportation in the city. Data about Timon's buses routes were taken from Moovit App. The Bus Journeys Dataset was also taken from STRANS/PMT and contains 45 090 bus trips for a 24 h interval for each bus line in 2006. The database describes single bus passenger's journey through the variables: Bus Route, Bus ID, Direction, Time at Origin, Origin Bus Stop and Destination Bus Stop.

## 3. Methods

In this section, we will present and briefly recount the details of the models we have used to estimate the impact of the introduction of the BRT system in Teresina and Timon, while also introducing the equations we have used to measure the accessibilities. As we will see, we have taken into account the journey to work distribution using both models and infrastructure to calculate the accessibilities.

### 3.1. The gravity model

In the gravity model approach, the flow from zone *i* to zone *j* is proportional to the opportunities, employment in this case, *E*_*j*_ in destination *j*, and to the demand in origin *i* (which is represented by the population) *P*_*i*_, and weighted by the cost function *f*(*c*_*ij*_). Given our approach, we consider the cost *c*_*ij*_ of going from *i* to *j* as the expected time of travel using the public transportation network (see appendix for details on the travel time calculations), and the function as being an exponential decay of the form *f*(*c*_*ij*_) = e^−*βc*_*ij*_^, where *β* is a parameter that has to be calibrated on data. Traditionally, to model the journey to work trip distribution, the total number of commuters is constrained (outflow), as are the employees arriving at work (inflow). This corresponds to the doubly constrained model, where the flows are described by the equation

3.1 $$T_{ij}^{\mathrm{dbl}}=A_{i}B_{j}P_{i}E_{\;j}\;{\text{e}}^{-\beta c_{ij}},$$

where *A*_*i*_ and *B*_*j*_ are two normalization constants that one has to solve iteratively. By imposing the constraints on the total outflow and on the total inflow

3.2 $$\sum_{j}T_{ij}=P_{i}\;\text{and}\;\sum_{i}T_{ij}=E_{j}$$

and following the procedure in [4], we get

3.3 $$A_{i}=\frac{1}{\sum_{k}B_{k}E_{k}\;{\text{e}}^{-\beta c_{ik}}}\;\text{and}\;B_{j}=\frac{1}{\sum_{k}A_{k}P_{k}\;{\text{e}}^{-\beta c_{kj}}}.$$

In its single-constrained version, where only the constraint on the outflow is kept, the flow from origin *i* and destination *j* in this case has the form

3.4 $$T_{ij}^{\mathrm{sng}}=Z_{i}P_{i}E_{j}\;{\text{e}}^{-\beta c_{ij}},$$

where *Z*_*i*_ is the normalization constant. Imposing the constraint on the outflow $\sum_{j}T_{ij}=P_{i}$ leads to

3.5 $$Z_{i}=\frac{1}{\sum_{k}E_{k}\;{\text{e}}^{-\beta c_{ik}}}.$$

In the next section, we will use both forms to reproduce the origin–destination matrix and to calculate the accessibilities.

### 3.2. The radiation model

The original radiation model [14] takes its inspiration from the intervening opportunities model [1]. In this approach, the probability of commuting between two units *i* and *j* depends on the number of opportunities between the origin and the destination, rather than on their distance. In its original formulation, the radiation model made use of the population in each zone, using it also as a proxy for employment. The flow between zone *i* and *j* is therefore quantified as

3.6 $$T_{ij}^{\mathrm{rad}}=T_{i}\frac{P_{i}P_{j}}{\left(P_{i}+P_{ij})(P_{i}+P_{j}+P_{ij}\right)},$$

where *P*_*ij*_ is the population in zones included in a radius of distance or travel time *d*_*ij*_, and excluding those of zones *P*_*i*_ and *P*_*j*_. These represent the opportunities between them, and *T*_*i*_ is the amount of commuters in *i*. As presented, the model in equation (3.6) was formulated to describe flows happening on large scales and the absence of parameters to calibrate makes the model hard to fit to smaller scales. For this reason, the form we have used is slightly different and following [21] we have added a normalization constant that takes into account the *finite size* of the system. Moreover, we have used the employment to characterize opportunities rather than the population. The flows between zones *i* and *j* are now described by

3.7 $$T_{ij}^{\mathrm{rad}}=\frac{P_{i}}{\left(1-P_{i}/P\right)}\frac{E_{i}E_{j}}{\left(E_{i}+E_{ij})(E_{i}+E_{j}+E_{ij}\right)},$$

where *P* is the total amount of population in the system, and where *E*_*ij*_ is the amount of employment between zones *i* and *j*. As previously said, for our analysis we have used the expected travel time to measure the cost of travelling from one zone to the another, therefore as in [34], *E*_*ij*_ here represents the opportunities within travel time *c*_*ij*_ from *i*.

We have also used the *extended* version of this model presented in [20] where a parameter *α* is introduced, and whose calibration makes the model adaptable to different spatial scales. The flow in this version is derived by combining the original radiation model with *survival analysis* [35] and in this context the probability of commuting from *i* to *j* is described by the equation

3.8 $$P(1\;|\;E_{i},E_{j},E_{ij})=\frac{\left[{\left(E_{i}+E_{j}+E_{ij}\right)}^{\alpha }-{\left(E_{i}+E_{ij}\right)}^{\alpha })](E_{i}^{\alpha }+1\right)}{\left[{\left(E_{i}+E_{ij}\right)}^{\alpha }+1][{\left(E_{i}+E_{j}+E_{ij}\right)}^{\alpha }+1\right]}.$$

The details of the calculations that lead to this form may be found in [20]. The flows in the extended version are the product of equation (3.8), the population in the origin zone *i*, and normalization term

3.9 $$T_{ij}^{\mathrm{ext}}=P_{i}\frac{P(1\;|\;E_{i},E_{j},E_{ij})}{\sum_{k}P(1\;|\;E_{i},E_{k},E_{ik})}.$$

One may note that as per our construction, equation (3.9) is constrained to meet the outflow $\sum_{k}T_{ik}=P_{i}$, but not the inflow $\sum_{k}T_{ki}\neq E_{i}$ which is correspondence in the singly constrained version of the gravity model.

### 3.3. Measures of accessibility

As mentioned in the introduction, in order to quantify the impact of the new infrastructure, we measure the accessibility predicted by the models before and after the introduction of the BRT, which is done by using both *c*^old^_*ij*_ and the updated cost matrix *c*^brt^_*ij*_. Of course, the variation of accessibility induced by changing the cost matrix will have an impact on the population and employment distributions. A zone with an increased accessibility will see the house price rise, which will affect the resident population and consequently the employment. Despite these *secondary* effects definitely playing an important role in the planning of such public interventions, they emerge as a result of complex nonlinear interactions and are therefore hard to predict. For this reason, their description goes beyond the scope of this work, where we have concentrated on the immediate impact of the transportation network.

Accessibility has become a central concept in physical planning in the past decades [36–40], and many different definitions exist which depend on the specific application. In general, as stated in [39], accessibility associates some measure of opportunity at a place with the cost of actually realizing that opportunity, or in other words as the cost of getting to some place traded off against the benefits received once that place is reached. In [39], two main types of accessibility are defined: type 1 takes into account the locational behaviour described through models, with infrastructure only implicitly considered; type 2 considers the physical infrastructure and some generalized measure of the *distance* from the zone of interest to all others. For a zone *i*, we define accessibility of type 1 as

3.10 $$A_{i}^{1}=\frac{\sum_{j}T_{ij}(1/c_{ij})}{\sum_{j}T_{ij}},$$

where *c*_*ij*_ is the cost of commuting from i→j (this is a measured quantity which depends on the infrastructure), and *T*_*ij*_ is the predicted flow and depends on the model used to calculate it. The accessibility in equation (3.10) quantifies the inverse of the average cost of commuting from the given area: high values of *A*_*i*_ correspond to the flows happening with low values of *c*_*ij*_. We define, as a simple measurement of the infrastructure, the accessibility of type 2 as

3.11 $$A_{i}^{2}=\frac{1}{N_{d}}\sum_{j}\frac{E_{j}}{c_{ij}},$$

where *E*_*j*_ are the opportunities (employment) in zone *j*, and *N*_*d*_ is the total number of destinations. Once again, equation (3.11) is telling us the average benefit–cost ratio for zone *i*. As we can see, there is no modelling involved in this measure, and we have only exploited the cost matrix and the opportunities distribution. Comparing equations (3.10) and (3.11) allows us to understand the information benefit of adding a modelling layer to the analysis, especially given the use of the different kinds of model we have applied.

## 4. Results

In the first part of this section, we calibrate the models on the origin and destination matrix. To do so, we used Sørensen's index which estimates the degree of similarity between the observed and modelled trips. This is how usually models are compared: the model that yields the highest value of the index is then considered the most fit to describe the system. As mentioned here, we compare models simulating a policy testing scenario, and therefore regardless of the results obtained during the calibration we then use the models to predict the variation of the accessibility of each zone after the introduction of the new BRT corridors. These results are found in the second part of the section. Finally, we study in detail the characteristics of the zones whose predictions obtained using the double-constrained gravity and the extended radiation models are similar, and those for which they are different, in search of systematic differences. We chose the two models given the great similarity in their results.

### 4.1. Sørensen's index calibration

As we have seen, the survey used to calibrate the models counts only 138 households, and therefore does not contain information on trips from every zone to every other. For each origin zone, we only have information on trips to a limited number of destinations. As done in previous comparisons [18–21], we calibrate our models using Sorensen's index [41], and exploit the dataset in [23]. The index is defined as

4.1 $$E^{S⊘\mathrm{rensen}}=\frac{2\sum_{i,j}\text{min}(T_{ij}^{\mathrm{model}},\;T_{ij}^{\mathrm{data}})}{\sum_{ij}T_{ij}^{\mathrm{model}}+\sum_{ij}T_{ij}^{\mathrm{data}}}$$

and by construction takes values between [0, 1], where *E*^Sørensen^ = 1 indicates that the flows in the data and in the model are identical, while *E*^Sørensen^ = 0 means the flows have no relation. Of course, *T*^model^_*ij*_ indicates the trip obtained using one of the models while *T*^data^_*ij*_ are the trips found in the data.

**Figure 2.**
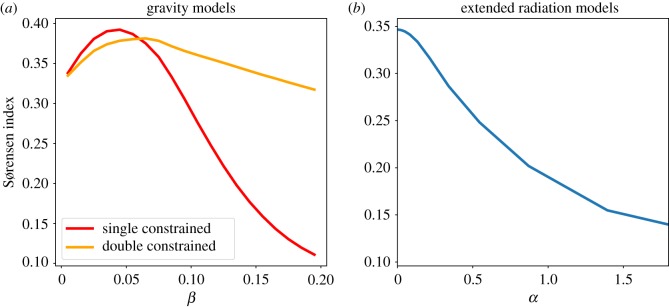
We show the behaviour of the Sørensen's index for (*a*) the gravity models and (*b*) the extended radiation model. If the gravity models exhibit a clear maximum for *β*_single_ = 0.045, *β*_double_ = 0.065, in the extended radiation context this is reached asymptotically for α→0.

In figure 2*a*, we show the form of Sørensen's index for the double- and single-constrained gravity models, and in figure 2*b* for the extended radiation model. For both versions of the gravity approach, the index presents a clear maximum with *β*_single_ = 0.045 and *β*_double_ = 0.065, values we have used to produce the results shown in this paper.

When repeating the calculation in equation (4.1) for the extended radiation model, we see how the maximum is asymptotically reached for α→0. Given that we are working at an intra-city scale this is not surprising, and in the electronic supplementary material of [20] (in §9) the authors have solved the model's form as equation (3.9) for this limit with these scales in mind. The equation that describes the flow from zone *i* to zone *j* for α→0 becomes

4.2 $$\lim_{\alpha \to 0}T_{ij}^{\mathrm{ext}}=P_{i}\frac{E_{j}/(E_{i}+E_{ij})}{\sum_{k}E_{k}/(E_{i}+E_{ik})}.$$

Perhaps surprisingly, the single-constrained gravity model is the one which performs the best with the index reaching *I*_sgl_ = 0.39, followed by the double-constrained where *I*_dbl_ = 0.38 and the extended radiation where *I*_ext_ = 0.34. The normalized radiation with no calibration process yielded a Sørensen index of *I*_rad_ = 0.22.

### 4.2. Accessibility variations after the introduction of the BRT

In order to quantify the impact of the introduction of the BRT, we have measured, for all zones in Teresina, the quantities in equations (3.10) and (3.11) using the cost matrices before the intervention *c*^old^_*ij*_ and after *c*^brt^_*ij*_. To explicitly study the variation introduced by the BRT system, we have then analysed the ratio of the two quantities

**Figure 3.**
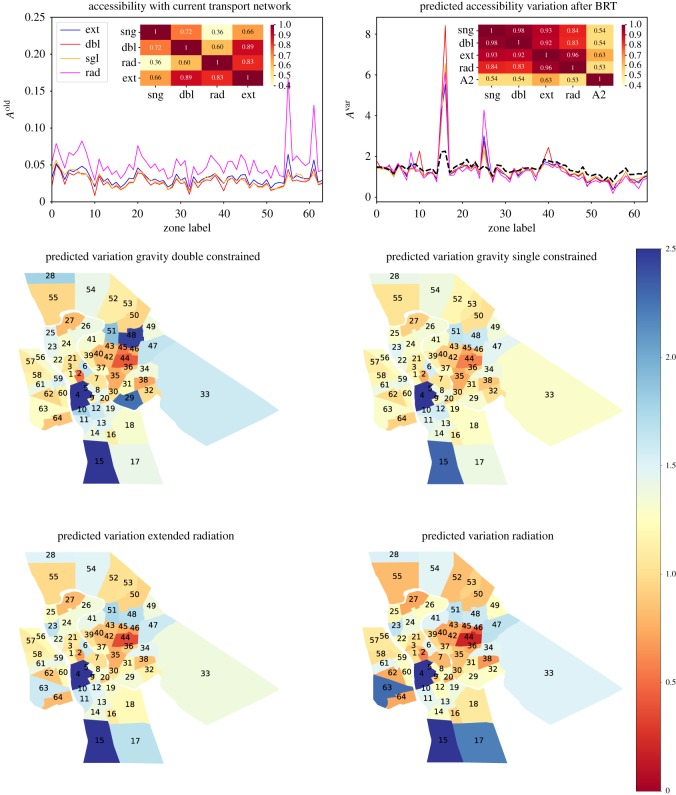
In this figure, we show the accessibility to employment with *A*^old^_*i*_ and *A*^var^_*i*_ = *A*^brt^_*i*_/*A*^old^_*i*_ on the top left and top right, respectively, for each zone of the city of Teresina and Timon. As noted in the legends of the figures, the red (double) and orange (single) curves show the results obtained from the gravity models, while the blue (extended) and magenta (normalized) curves represent the results obtained with the radiation models. The black curve in the accessibility variation is the variation measured with respect to accessibility of type 2. In the insets, we present the *R*^2^ values between the various curves. The maps in the bottom panels present the spatial distribution of *A*^var^ obtained with all the models.

4.3 $$A_{i}^{\mathrm{var}}=\frac{A_{i}^{\mathrm{brt}}}{A_{i}^{\mathrm{old}}},$$

so that the zones with *A*^var^_*i*_ > 0 are predicted to benefit from the intervention and vice versa. We repeat this process using the gravitational and radiation approach. The results are summarized in figure 3, where we show in detail all the quantities we have discussed and the spatial distribution of the predicted impact on the city's zoning system. In the top panel, we have shown *A*^old^ and *A*^var^ calculated with the various models. Given the difference in the characteristic values between the accessibilities of type 1 and 2, we only compare their predictions on the *A*^var^, where the variation of type 2 is represented by a black curve. We will refer to *A*^dbl^_*i*_, *A*^sng^_*i*_, *A*^ext^_*i*_ and *A*^rad^_*i*_ for the accessibilities calculated using the double-constrained gravity, single-constrained gravity and the extended and normalized radiation model, respectively.

What immediately catches the eye is the substantial agreement between the *A*^ext^_*i*_ (blue curve) and *A*^dbl^_*i*_ (red curve) before the BRT introduction. The *R*^2^ values in the insets confirm this impression, with higher values between the two models than that measured between *A*^dbl^_*i*_ and *A*^sng^_*i*_. This is quite unexpected, especially considering the fact that per construction, the extended radiation model is a singly constrained model. Very similar results apply when calculating the accessibilities on the new cost matrix *c*^brt^_*ij*_, these have not been shown to avoid redundant figures.

Looking at the accessibility variations *A*^var^_*i*_, it is clear that all models predict two main peaks, one for zones 17 and 16, and another for zone 26. The first peak can be seen analysing both types of accessibility, *A*^1^_*i*_ and *A*^2^_*i*_ (black curve), while the peak in zone 26 is not captured by the measure of the infrastructure. The BRT map in figure 1 shows that a new BRT stop is planned near zone 26, so a spike in its accessibility is indeed reasonable. This seems to suggest that the behavioural layer introduced through the modelling of the flows adds more information to the simple analysis of infrastructure. The variation of the accessibilities of type 1 predicted by all models has a high *R*^2^ value (*R*^2^ > 0.8), while the correlation between the models and *A*^2^ is significantly lower.

To understand the spatial configuration of the results, we show heat maps of the *A*^var^ on the map of the city in figure 3. The maps show that if the zones in the city centre are expected to benefit from the new transport network, the main benefits are located in the south zones 16 and 17, and in the centre north zones 26 and 27. We can see from the BRT map in figure 1 that these zones are close to where new bus stops will be positioned. The zones in the north of the city, despite being close to the new stops and served by a BRT, are expected to generate longer journeys to work, and therefore their accessibility will be lower. The zones in the district of Timon are not included in the BRT intervention, and it is therefore not surprising to find that their employment accessibilities decrease according to all the three models.

In general, we can say that the predictions obtained with the models are in good agreement. This is clear by looking at the high *R*^2^ values obtained when comparing the curves, and it is especially true for the doubly constrained and the extended radiation models. Moreover, the normalized radiation's predictions are in great agreement with the other models, which was not expected considering the absence of a calibration process, and a low Sørensen index. To conclude, we can say that the model's predictions are coherent and, bearing in mind the spatial distribution of the BRT stops and corridors, also reasonable, and that the accessibility of type 1 seems a more appropriate measure for this study than the measure of type 2.

### 4.3. The doubly constrained gravity versus the extended radiation model

As portrayed in the introduction, one of the main objectives of this work is to analyse similarities and differences in the predictions made using the two approaches. Despite the great agreement found between the doubly constrained and the extended radiation models, several zones show contradictory results. We will now look into the properties of these specific zones, and check if there are similarities among them. We do this by comparing the predictions on the accessibility after the BRT introduction, *A*^ext^_*i*_ and *A*^dbl^_*i*_, by analysing the difference in the rank of each zone, namely

**Figure 4.**
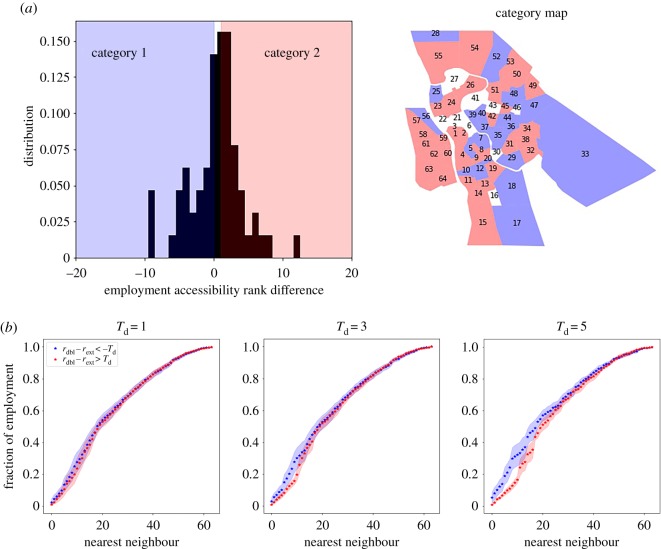
In (*a*), we show the distribution of the values of the Δ*r*, and a map of the spatial distribution of the zones. The blue zones highlight zones in category 1, the white area zones in category 0 and the red in category 2. In (*b*), we show the average distance to employment of zones in categories 1 and 2. For bigger values of *T*_d_, we compare zones of increasing difference in the performance using the two models. The shaded areas represent the standard deviation of the distribution.

4.4 $$\Delta r_{i}=r_{i}^{\mathrm{dbl}}-r_{i}^{\mathrm{ext}},$$

where *r*^dbl^_*i*_ is the rank of zone *i* using the gravity and *r*^ext^_*i*_ is the rank obtained using the radiation model. If Δ*r*_*i*_ > 0, this implies that *r*^dbl^_*i*_ > *r*^ext^_*i*_, which means that the accessibility of zone *i* ranks higher if we use the gravitational model to calculate the accessibilities, and vice versa. The distribution of the quantity in equation (4.4) calculated using the two models is found in the top panel of figure 4. The red and blue areas highlight the zones for which the predictions vary (∥Δ*r*∥ > 0), which are those whose characteristics we want to analyse.

By introducing a threshold on the rank difference, we can divide the zone into three categories:

— Category 1: zones that rank higher when using the extended radiation model of a quantity *T*_d_: Δ*r*_*i*_ < – *T*_d_ (blue in the figures).
— Category 2: zones that rank higher when using the double-constrained model of a quantity: −*T*_d_: Δ*r*_*i*_ > *T*_d_ (red in the figures).
— Category 3: zones whose rank is similar when using the two models: *T*_d_ < Δ*r*_*i*_ < *T*_d_ (white in the figures).

Of course for increasing values of *T*_d_, categories 1 and 2 contain zones of whose predictions vary considerably using the two models.

We have studied several characteristics of the zones that belong to each category: the population and employment distribution, their spatial distribution and their distance to employment. The population and employment distributions are completely comparable in all three categories, and for this reason we have not shown it in the figures. Furthermore, no precise spatial pattern emerges when projecting these categories on the city's zoning system (map in figure 4*a*). But, an interesting difference emerges when quantifying the distance to employment of the different categories, as we can see from figure 4*b*. What emerges is a clear tendency of the zones of category 1 to be closer to the opportunities (blue curves) than those of category 2 (red curves). By looking at the figure, it is clear how the blue curve tends to increase faster than the red one. Zones in category 1 tend to be surrounded by zones rich in employment, while category 2 neighbouring zones have less employment. This seems to imply that the extended radiation tends to give a higher weight to closer neighbours than the double-constrained gravity model, and probably depends on the different way the two models handle distance (metric vs topological). Moreover we can see from figure 4 how this tendency increases when increasing the difference threshold *T*_d_.

## 5. Discussion

In this work, we used two different approaches to test the impact of a new transportation system. In doing so, we achieved two results: on the one hand, we were able to identify which zones would benefit the most from such an infrastructure project by increasing their accessibility to jobs and services; and on the other, we were able to compare the performance of the gravity and the radiation models on real data. Interestingly, the agreement between the results obtained with all models is outstanding. This may suggest that the detailed reproduction of the observed distribution is not crucial in a city planning context with respect to measuring accessibility variations at the scale we are working at. Furthermore, we have compared the estimated impact on the accessibilities, using the type 1 and 2 definitions.

The type 1 accessibility seemed to be in better agreement with the expected impact. By looking at figure 3, we see that the zones with the highest estimated improvement, zones 15, 16 and 26, have low starting accessibilities and are positioned next to a BRT stop. The modelling of flows does seem to add information that the policy maker cannot extract simply by observing the differences in the infrastructure and the trip durations. With this in mind, the models that seem to better describe mobility are the extended radiation and the doubly constrained gravity model. We have seen how, despite considerable agreement, the predictions obtained by the two models differ for those zones with many opportunities around them and those with very few. Indeed, we have seen that the zones with a neighbourhood rich in opportunities perform better with the gravity model, and vice versa. This is an interesting result, which is potentially useful in urban planning scenarios. The results we present do not take into consideration exogenous changes to the system as an indirect consequence of introducing the BRT network. This is because accessibility, population and employment distribution are inter-dependent, and nonlinear relationships emerge feeding back on each other. In this work, we have not taken into account these externalities and have only described how the new transportation network would impact the accessibility distribution. We leave the rest of the analysis to future work. That said, we may conclude by saying that these preliminary results show that the radiation model in its extended version could be a valid alternative to study urban mobility and test new transportation networks. More research on this topic would help us better understand how the two models could support each other.

## Data accessibility

All the data used in this work can be found in the public repository, https://github.com/ducciopiovani/Data-on-Teresina-, where we have stored the cost matrices and the origin and destination matrix in three separate files.

## Appendix A

**A.1. Cost function calculations**

The effects of the new transportation network on trip distribution have been measured by calculation of the generalized costs of travel, for each pair of zones, i→j. To do so, we had to calculate two cost matrices, one before {*c*^old^_*ij*_} and one after {*c*^brt^_*ij*_} the introduction of the BRT. Each element of the matrices indicates the time necessary to travel from zone *i* to zone *j* using public transportation. We have collected the travel times of the old transportation network from Google Maps Directions, using the API, which provides a service to calculate directions between requested locations considering available modes of transportation. The API request takes as input origins and destinations, which were taken from zone centroid coordinates, and gives an estimate of the expected time of travel. The results were stored in the origin destination cost matrix *c*^old^. On the other hand, the travel times after the BRT introduction had to be predicted. To estimate new routes, we have built a model using ArcGIS Spatial Analyst Extension for the existing bus network with the current local bus GTFS (given by the Transport Authority of Teresina), and calibrated with real travel time data and the predicted travel time between pairs of zones was calculated. Further developments for the BRT systems were introduced in the network (BRT GTFS), and the predicted travel time between pairs of zones was calculated. In this case, the results were stored in the origin destination cost matrix *c*^brt^_*ij*_.
